# Evaluating the prognostic role of glucose-to-lymphocyte ratio in patients with metastatic renal cell carcinoma treated with tyrosine kinase inhibitors in first line: a study by the Turkish Oncology Group Kidney Cancer Consortium (TKCC)

**DOI:** 10.1007/s12094-024-03813-w

**Published:** 2025-01-15

**Authors:** Hatice Bolek, Omer Faruk Kuzu, Elif Sertesen Camoz, Saadet Sim, Serhat Sekmek, Hilal Karakas, Selver Isık, Murat Günaltılı, Aysun Fatma Akkus, Deniz Tural, Cagatay Arslan, Sema Sezin Goksu, Ozlem Nuray Sever, Nuri Karadurmus, Cengiz Karacin, Mehmet Ali Nahit Sendur, Emre Yekedüz, Yuksel Urun

**Affiliations:** 1 Department of Medical Oncology, Ankara University School of Medicine, 06590 Ankara, Türkiye; 2 Ankara University Cancer Institute, Ankara, Türkiye; 3 Department of Medical Oncology, Gulhane Training and Research Hospital, University of Health Sciences, Ankara, Türkiye; 4 Department of Medical Oncology, Dr Abdurrahman Yurtaslan Oncology Training and Research Hospital, University of Health Sciences, Ankara, Türkiye; 5 Department of Medical Oncology, Ege University School of Medicine, Izmir, Türkiye; 6 Department of Medical Oncology, Bilkent City Hospital, Ankara, Türkiye; 7 Department of Medical Oncology, Marmara University School of Medicine, Istanbul, Türkiye; 8 Department of Medical Oncology, Cerrahpasa School of Medicine, Istanbul, Türkiye; 9 Department of Medical Oncology, Trakya University School of Medicine, Edirne, Türkiye; 10 Department of Medical Oncology, Bakirkoy Dr. Sadi Konuk Training and Research Hospital, University of Health Sciences, Istanbul, Türkiye; 11 Medical Point Hospital, Izmir University of Economics, Izmir, Türkiye; 12 Department of Medical Oncology, Akdeniz University School of Medicine, Antalya, Türkiye; 13 Department of Medical Oncology, Kartal Dr. Lutfi Kirdar City Hospital, Istanbul, Türkiye; 14 Department of Medical Oncology, Dana-Farber Cancer Institute, Boston, MA USA

**Keywords:** Renal cell carcinoma, Glucose-to-lymphocyte ratio, GLR, Tyrosine kinase inhibitor

## Abstract

**Purpose:**

Identifying prognostic indicators for risk stratification in metastatic renal cell carcinoma (mRCC) is crucial for optimizing treatment strategies and follow-up plans. This study aims to investigate the prognostic role of the glucose-to-lymphocyte ratio (GLR) in patients with mRCC receiving tyrosine kinase inhibitors (TKIs) as first-line therapy.

**Methods:**

A retrospective cohort study was conducted using data from the Turkish Oncology Group Kidney Cancer Consortium Database. GLR was calculated by dividing the fasting glucose (mmol/L) by the lymphocyte count (×10^9^/L). We categorized patients into two categories based on their median GLR level.

**Results:**

The analysis included a total of 598 patients. We found that progression-free survival (PFS) was significantly longer in the GLR-low group, with a median PFS of 15.05 months (95% CI 12.7–17.4) compared to 7.79 months (95% CI 6.6–9.0) in the GLR-high group (*p* < 0.001). Multivariate analysis identified GLR as an independent risk factor for poor PFS (HR 1.39, 95% CI 1.12–1.72; *p* = 0.003). Overall survival (OS) was also significantly longer in the GLR-low group, with a median OS of 38.47 months (95% CI, 30.9–46.0) compared to 24.15 months (95% CI 18.0–30.2) in the GLR-high group (*p* = 0.001). GLR was an independent predictor for OS in multivariate analysis (HR 1.45, 95% CI 1.12–1.86; *p* = 0.004).

**Conclusion:**

The GLR can be a valuable prognostic marker for glucose metabolism and systemic inflammatory status in this patient population. Our research highlights the potential prognostic value of GLR in patients with mRCC receiving TKIs, indicating its potential as a useful tool for clinical decision-making.

## Introduction

### Purpose

Renal cell carcinoma (RCC) accounts for 2.2% of all new cancer cases, with an estimated 434,419 new cases and 155,702 deaths globally in 2022 [1]. 5-year survival rates are 93% for local, early-stage disease, 75% for locally advanced disease, and 18% for metastatic disease [2]. Therefore, metastatic RCC (mRCC) has a poor prognosis, underscoring the need for practical risk stratification tools. Establishing independent prognostic indicators for risk stratification in mRCC could assist in tailoring optimal treatment strategies and follow-up plans for patients with mRCC. The International mRCC Database Consortium (IMDC) prognostic risk model, which considers six clinical and laboratory parameters, is the current clinical standard for risk stratification and influences treatment decisions in mRCC [3, 4]. However, additional biomarkers and clinical factors that could improve prognosis accuracy and guide personalized therapy require ongoing research to refine and enhance the prognostic models.

Chronic, dysregulated inflammation has been linked to an increased risk of malignancies as well as the progression of the cancer [5–9]. Furthermore, increasing evidence suggests that the inflammatory tumor microenvironment plays a role in determining treatment’s therapeutic efficacy [10, 11]. Consequently, biomarkers or cells associated with inflammation may provide valuable insights into the cancer prognosis, offering potential indicators of disease progression and response to treatment. Understanding these inflammatory markers could enhance our ability to predict outcomes and effectively tailor therapeutic strategies. The prognostic role of several scores and ratios, which include inflammatory markers or cells related to inflammation, were investigated in mRCC. The Meet URO score, which combines the IMDC prognostic classification with two additional factors—the pre-treatment presence of bone metastases and peripheral blood neutrophil-to-lymphocyte ratio (NLR)—demonstrated effective stratification of pretreated mRCC patients receiving immunotherapy [12, 13]. The Modified Glasgow Prognostic Score, another inflammatory marker based on two routine laboratory parameters—C-reactive protein (CRP) and albumin—has been shown to have prognostic and predictive value in mRCC [14, 15]. Additionally, studies have demonstrated the prognostic value of numerous ratios in mRCC, including the platelet-to-lymphocyte ratio, the lymphocyte-to-monocyte ratio, the CRP-to-albumin ratio, and the NLR [16–20].

A novel inflammatory marker, glucose-to-lymphocyte ratio (GLR), is prognostic in patients with RCC undergoing nephrectomy [21, 22]. However, no study in the literature has investigated whether GLR impacts survival outcomes in metastatic RCC. This study aims to investigate the prognostic role of GLR in patients with mRCC using TKIs as a first-line treatment.

## Methods

We conducted a retrospective cohort study by extracting patient data from the Turkish Oncology Group Kidney Cancer Consortium (TKCC) Database, which includes patients with mRCC from 13 cancer centers in Turkey. The study included patients who had a diagnosis of mRCC, older than 18 years of age, and who received TKI monotherapy as first-line treatment. The study excluded patients whose lymphocyte count or fasting glucose level was not available within 15 days before the start of TKI therapy. For patients with multiple values, the value closest to the beginning of treatment was used.

We extracted demographic (e.g., date of birth, gender) and clinical data (e.g., the date of mRCC diagnosis, the initial date of systemic treatment in the metastatic setting, Eastern Cooperative Oncology Group (ECOG) performance scores, laboratory findings, and dates of progression and death from the TKCC database. The glucose-to-lymphocyte ratio (GLR) was calculated by dividing the fasting glucose (mmol/L) by the lymphocyte count (×10^9^/L). We categorized patients into two categories based on their median GLR level. "GLR-high" refers to patients with a GLR level that exceeds the median GLR of the whole cohort, while "GLR-low" refers to patients with a GLR level that is equal to or lower than the median GLR of the whole cohort. PFS was defined as the time from the start of first-line TKI treatment to disease progression or death. OS was defined as the time from the start of first-line TKI treatment to death.

All statistical analyses were performed using the IBM SPSS Statistics 24.0 software package. Continuous variables were described as medians [interquartile range (IQR)] and categorical variables as percentages. The Chi-square test was used to compare categorical variables, while the Mann–Whitney *U* test or Student’s *t*-test was used to compare continuous variables. Survival curves and rates were estimated using the Kaplan–Meier method. Multivariate analyses were performed using variables with a *p* value of ≤ 0.20 in the univariate analyses. Cox regression analyses were conducted to perform multivariable analyses and calculate hazard ratios (HRs) with 95% confidence intervals (CIs). All reported *p* values were two-sided, and *p* values < 0.05 were regarded as statistically significant.

Ethical approval number: Ankara University Faculty Ethics Committee, I09-701-24.

## Results

A total of 598 patients with a median age of 58.9 (IQR = 14.2) years were included in the study. Of these patients, 433 (71.7%) were male, and 509 (85.1%) patients have ECOG 0 or 1 performance status. Overall, 473 (79.1%) patients were diagnosed as clear cell RCC, while 83 (13.9%) of them had non-clear cell pathology, and 425 (71.1%) patients underwent nephrectomy either before or after the initiation of their treatment. Most used TKIs were sunitinib (56.7%) and pazopanib (40.1%).

The median GLR value was 3.64. Baseline characteristics were almost similar in GLR-low and GLR-high groups. However, the GLR-low group had a higher percentage of patients under 65 years of age (77.6 vs. 65.9%, *p* = 0.002) and those with an ECOG performance status of 0–1 (91 vs. 79.2%, *p* < 0.001). The rate of patients with bone metastasis was higher in the GLR-high group than in the GLR-low group (45.2 vs. 31.8%, *p* = 0.001). All baseline characteristics are shown in Table 1.

**Table 1:** General characteristics of the patients

	All patients *N* = 598 (%)	GLR-high patients *N* = 299 (%)	GLR-low patients *N* = 299 (%)	*p*
Age (years)				
Median (IQR)	58.98 (14.2)	60.68 (15.1)	57.22 (13.7)	**0.001**
<65	429 (71.7)	197 (65.9)	223 (77.6)	**0.002**
≥65	169 (28.3)	102 (34.1)	67 (22.4)	
Gender				
Male	433 (72.4)	212 (70.9)	221 (73.9)	0.464
Female	165 (27.6)	87 (29.1)	78 (26.1)	
ECOG PS				
0–1	509 (85.1)	237 (79.2)	272 (91)	** < 0.001**
2–3–4	77 (12.9)	57 (19.1)	20 (6.7)	
Unknown	12 (2)	5 (1.7)	7 (2.3)	
Diabetes mellitus				
Yes	129 (21.6)	75 (25.1)	54 (18.1)	**0.037**
No	464 (77.6)	221 (73.9)	243 (81.3)	
Unknown	5 (0.8)	3 (1)	2 (0.6)	
Histological type				
Clear	473 (79.1)	239 (80)	234 (78.3)	0.634
Non-clear	83 (13.9)	39 (13)	44 (14.7)	
Unknown	42 (7)	21 (7)	21 (7)	
Grade				
Grade 1–2	108 (18.1)	52 (17.4)	56 (18.7)	1.00
Grade 3–4	288 (48.2)	140 (46.8)	148 (49.5)	
Unknown	202 (33.7)	107 (35.8)	95 (31.8)	
Sarcomatoid feature				
Yes	66 (11)	28 (9.4)	38 (12.7)	0.353
No	388 (64.9)	190 (63.5)	198 (66.2)	
Unknown	144 (24.1)	81 (27.1)	63 (21.1)	
Nephrectomy				
Yes	425 (71.1)	199 (66.5)	226 (75.6)	**0.022**
No	167 (27.9)	96 (32.1)	71 (23.7)	
Unknown	6 (1)	4 (1.4)	2 (0.7)	
IMDC risk group				
Favorable	99 (16.5)	43 (14.4)	56 (18.7)	0.433
Intermediate	330 (55.2)	166 (55.5)	164 (54.8)	
Poor	135 (22.6)	69 (23.1)	66 (22.1)	
Unknown	34 (5.7)	21 (7)	13 (4.3)	
Metastatic sites				
Bone	230 (38.5)	135 (45.2)	95 (31.8)	**0.001**
Lung	408 (68.2)	196 (65.6)	212 (70.9)	0.188
Liver	110 (18.4)	58 (19.4)	52 (17.4)	0.598
LN/soft tissue	359 (60)	184 (61.5)	175 (58.5)	0.504
CNS	46 (7.7)	25 (8.4)	21 (7)	0.646
First line treatment				
Sunitinib	339 (56.7)	160 (53.5)	179 (59.9)	0.247
Pazopanib	240 (40.1)	127 (42.5)	113 (37.8)	
Cabozantinib	12 (2)	8 (2.7)	4 (1.3)	
Sorafenib	5 (0.8)	2 (0.7)	3 (1)	
Axitinib	2 (0.3)	2 (0.7)	0	
Prior cytokine treatment				
Yes	179 (29.9)	78 (26.1)	101 (33.8)	**0.049**
No	419 (70.1)	221 (73.9)	198 (66.2)	

*CNS* central nervous system, *ECOG* Eastern Cooperative Oncology Group, *GLR* glucose-to-lymphocyte ratio, *IMDC* International Metastatic Renal Cell Carcinoma Database Consortium, *IQR* interquartile range, *LN* lymph node, *PS* performance status

*p* values < 0.05 were regarded as statistically significant

**Fig. 1:**
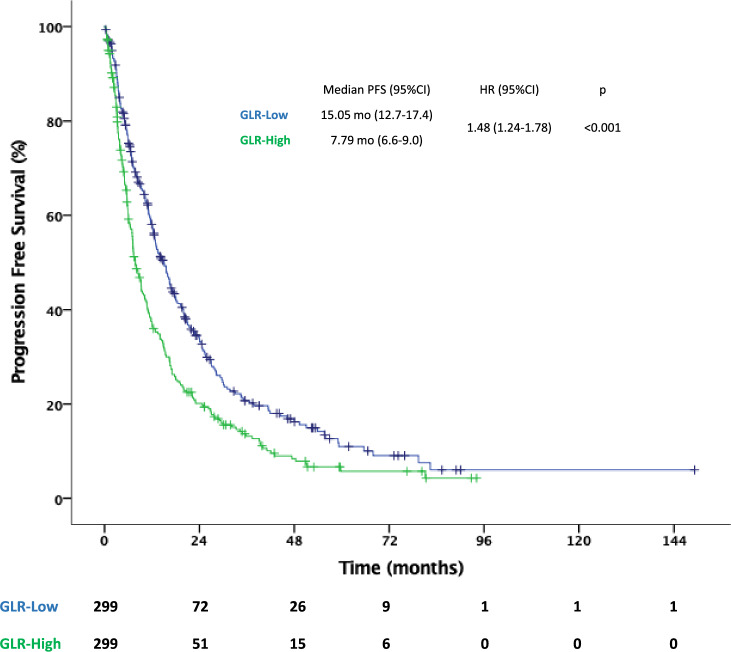
Kaplan–Meier curve for progression free survival. Abbreviations: *CI* confidence interval, *GLR *glucose-to-lymphocyte ratio, *HR* hazard ratio, *PFS* progression free survival

**Fig. 2:**
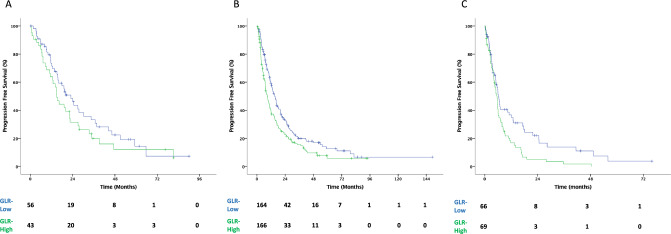
Kaplan–Meier curve for progression free survival in patients with IMDC favorable (**A**), intermediate (**B**) and poor (**C**) risk groups. Abbreviations: *CI* confidence interval, *GLR* glucose-to-lymphocyte ratio, *HR* hazard ratio, *PFS* progression free survival

Median PFS was 11.17 (95% CI 9.8–12.5) months for all population. PFS was significantly longer in the GLR-low group than in the GLR-high group (15.1 (95% CI 12.7–17.4) vs. 7.79 (95% CI 6.6–9) months, *p* < 0.001). Among patients with IMDC-favorable risk, the difference in PFS between the two groups was not statistically significant (22.9 vs. 11.9 months for the GLR-low and -high groups, respectively; *p* = 0.209). However, the median PFS was longer in the GLR-low group than in the GLR-high group among patients with IMDC intermediate (15 vs. 8.7 months, *p* = 0.030) and poor (6.2 vs. 5.5 months, *p* = 0.009) risk scores (Figs. 1 and 2). Multivariate Cox regression analysis revealed that GLR was consistently an independent predictor for PFS (GLR-high vs. GLR-low; HR 1.39, 95%CI 1.12–1.72; *p* = 0.003) after adjusting for confounding factors, such as age, ECOG performance status, sarcomatoid features, previous nephrectomy, IMDC risk groups, and bone metastasis (Table 2).

**Table 2:** Univariate and multivariate analysis for predictors of progression free survival

Variable	Univariate	*p*	Multivariate	*p*
	HR (95%CI)		HR (95%CI)	
Age				
<65	1		1	
≥65	1.50 (1.23–1.83)	**<0.001**	1.33 (1.05–1.68)	**0.016**
Gender				
Female	1		–	
Male	1.07 (0.87–1.31)	0.536	–	–
ECOG PS				
0–1	1		–	
2–3–4	1.77 (1.37–2.28)	**<0.001**	–	–
Diabetes mellitus				
No	1		–	
Yes	1.02 (0.82–1.28)	0.842	–	–
Histological type				
Clear	1		–	
Non-clear	1.05 (0.81–1.37)	0.707	–	–
Grade				
Grade 1–2	1		–	
Grade 3–4	0.99 (0.78–1.27)	0.957	–	–
Sarcomatoid feature				
No	1		1	
Yes	1.36 (1.03–1.79)	**0.038**	1.40 (1.04–1.89)	**0.029**
Nephrectomy				
No	1		1	
Yes	0.52 (0.43–0.64)	**<0.001**	0.65 (0.49–0.86)	**0.002**
IMDC risk group				
Favorable	1		1	
Poor	2.61 (1.94–3.51)	**<0.001**	1.91 (1.33–2.73)	**<0.001**
Bone metastasis				
No	1		1	
Yes	1.52 (1.26–1.83)	**<0.001**	1.27 (1.03–1.58)	
Prior cytokine				
No	1		–	
Yes	0.99 (0.82–1.21)	0.973	–	–
GLR				
GLR-low	1		1	
GLR-high	1.48 (1.24–1.78)	**<0.001**	1.39 (1.12–1.72)	**0.003**

*Abbreviations: CI* confidence interval, *ECOG* Eastern Cooperative Oncology Group, *GLR* glucose-to-lymphocyte ratio, *HR* hazard ratio, *IMDC* International Metastatic Renal Cell Carcinoma Database Consortium, *PS* performance status

Multivariate analyses were performed using variables with a *p* value of ≤ 0.20 in the univariate analyses.

*p* values < 0.05 were regarded as statistically significant in multivariate analysis

**Fig. 3:**
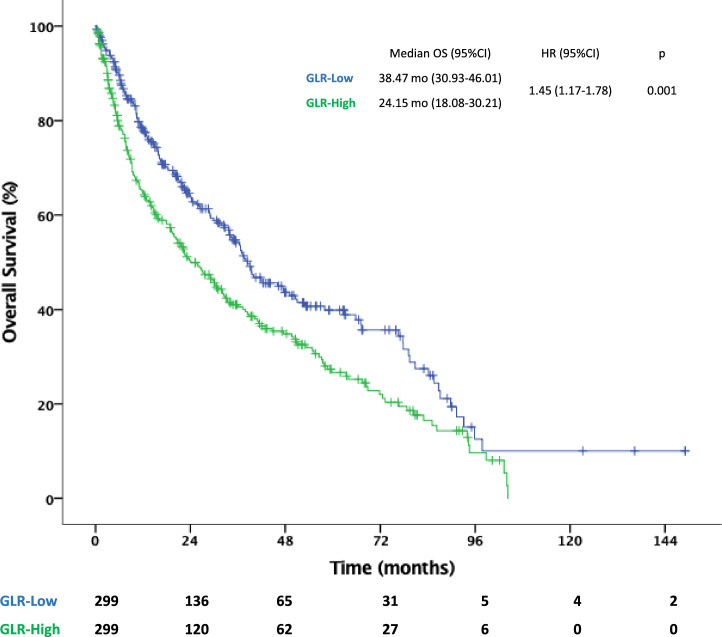
Kaplan–Meier curve for overall survival. *CI* confidence interval, *GLR* glucose-to-lymphocyte ratio, *HR* hazard ratio, *OS* overall survival

**Fig. 4:**
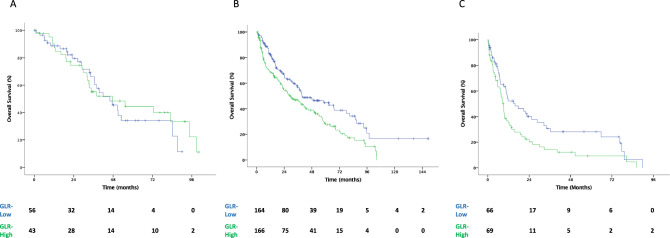
Kaplan–Meier curve for overall survival in patients with IMDC favorable (**A**), intermediate (**B**) and poor (**C**) risk groups. *CI* confidence interval, *GLR* glucose-to-lymphocyte ratio, *HR* hazard ratio

Median OS was 32.78 (95% CI 27.8–37.7) months for all population. OS was significantly longer in the GLR-low group than in the GLR-high group [38.47 (95% CI 30.9–46) vs. 24.15 (95% CI 18–30.2) months, *p* = 0.001]. Among patients with IMDC-favorable risk, the difference in OS between the two groups was not statistically significant (46.1 vs. 47.5 months for the GLR-low and -high groups, respectively; *p* = 0.653). Yet, the median OS was longer in the GLR-low group than in the GLR-high group among patients with IMDC intermediate (39.5 vs. 29.1 months, *p* = 0.003) and poor (15.8 vs. 8.9 months, *p* = 0.016) risk scores (Figs. 3 and 4). Multivariate Cox regression analysis showed that GLR was an independent predictor for OS (GLR-high vs. GLR-low; HR 1.45, 95% CI 1.12–1.86; *p* = 0.004) after adjusting for confounding factors, such as age, ECOG performance status, sarcomatoid features, previous nephrectomy, IMDC risk groups, bone metastasis, and prior cytokine use (Table 3).

**Table 3:** Univariate and multivariate analysis for predictors of overall survival

Variable	Univariate	*p*	Multivariate	*p*
	HR (95% CI)		HR (95% CI)	
Age				
<65	1		1	
≥65	1.65 (1.32–2.07)	**<0.001**	1.51 (1.16–1.98)	**0.002**
Gender				
Female	1		–	
Male	0.90 (0.71–1.13)	0.347	–	–
ECOG PS				
ECOG 0–1	1		1	
ECOG 2–3–4	2.45 (1.86–3.22)	**<0.001**	1.95 (1.36–2.79)	**<0.001**
Diabetes mellitus				
No	1		–	
Yes	1.06 (0.82–1.37)	0.646	–	–
Histological type				
Clear	1		–	
Non-clear	1.22 (0.89–1.65)	0.221	–	–
Grade				
Grade 1–2	1		–	
Grade 3–4	1.07 (0.81–1.42)	0.628	–	–
Sarcomatoid feature				
No	1		1	–
Yes	1.40 (1.02–1.92)	**0.048**	1.36 (0.96–1.93)	0.080
Nephrectomy				
No	1		1	
Yes	0.56 (0.45–0.70)	**<0.001**	0.62 (0.45–0.85)	**0.003**
IMDC risk group				
Favorable	1		1	
Poor	2.96 (2.10–4.15)	**<0.001**	2.28 (1.49–3.49)	**<0.001**
Bone metastasis				
No	1		1	
Yes	1.54 (1.25–1.90)	**<0.001**	1.35 (1.05–1.73)	**0.018**
Prior cytokine				
No	1		1	
Yes	1.31 (1.05–1.63)	**0.019**	1.29 (0.97–1.71)	0.083
GLR				
GLR-low	1		1	
GLR-high	1.45 (1.17–1.78)	**0.001**	1.45 (1.12–1.86)	**0.004**

*Abbreviations: CI* confidence interval, *ECOG* Eastern Cooperative Oncology Group, *GLR* glucose-to-lymphocyte ratio, *HR* hazard ratio, *IMDC* International Metastatic Renal Cell Carcinoma Database Consortium, *PS* performance status

Multivariate analyses were performed using variables with a *p* value of ≤ 0.20 in the univariate analyses.

*p* values < 0.05 were regarded as statistically significant in multivariate analysis

## Discussion

In this study, we investigate the prognostic role of GLR in patients with mRCC. Patient and disease characteristics such as age, gender, histological type, and IMDC risk category distribution are consistent with the general literature [23–25]. This study showed that high GLR levels were independent prognostic factors for PFS and OS in patients with mRCC who were treated with TKIs in the first-line setting. The predictive role of preoperative GLR was shown in several cancer types [21, 26–28]. Higher GLR levels were found to be an independent prognostic factor for OS and cancer-specific survival in patients with RCC who underwent laparoscopic nephrectomy [22]. The number of patients with metastatic disease was limited in these studies, and they did not assess the treatment outcomes. Using prognostic markers in cancer patients paves the way for more targeted and individualized approaches to cancer treatment, ultimately improving patients’ quality of life and treatment outcomes.

High serum glucose levels have been linked to an increased incidence of cancer as well as higher mortality rates among cancer patients [29–32]. Hyperglycemia indirectly affects cancer cells by increasing circulating levels of insulin and insulin-like growth factor 1, which are associated with enhanced tumor cell proliferation and reduced apoptosis [30, 33]. Hyperglycemia also induces a pro-inflammatory state, elevates oxidative stress levels, and activates several oncogenic pathways contributing to tumor development and proliferation [34, 35]. Circulating levels of proinflammatory cytokines such as interleukin (IL)−6, IL-18, and tumor necrosing factor-α (TNF-α) are revealed to be elevated by acute hyperglycemia-induced oxidative stress [36]. Both increased levels of IL-6 and IL-8 were associated with poor outcomes in RCC and lower response and resistance to sunitinib and pazopanib treatment [37–39]. Besides this, hyperglycemia causes activation or overexpression of some protooncogenes, including hypoxia-inducible factor-1α (HIF1-α), AKT, mammalian target of rapamycin (mTOR), and c-myc [40–42]. Activation of the AKT-mTOR pathway results in elevated expression of HIF-2α and enhanced lysosomal biosynthesis, which may cause TKI resistance [43, 44].

Moreover, epigenetic alterations in cancer-related genes may arise due to the prolonged exposure of primary cancer cells to high blood sugar levels. These alterations persist even after returning to normal blood sugar levels, potentially contributing to more aggressive tumor growth [45–47]. High blood glucose levels cause microRNA-16 (miR-16) expression to be downregulated by the overexpressed Myc gene [48]. Additionally, hypoxia and hypoxia-inducible factor-1α (HIF1-α), which play a critical role in the pathogenesis of RCC, lead to a decrease in miR-16 levels [49, 50]. Dejean et al. have demonstrated that downregulation of miR-16 leads to increased vascular endothelial growth factor (VEGF) expression, angiogenesis, and growth in anaplastic large-cell lymphoma [50]. The VEGF pathway is crucial in RCC, driving tumor angiogenesis and progression, making it one of the key targets for therapeutic intervention. As a result, hyperglycemia is linked to a variety of pathways related to cancer progression and drug resistance.

Lymphocytes play a vital role within the tumor microenvironment, serving as a critical defense mechanism against tumor growth and spread. The level of lymphocytes in peripheral blood is considered an indicator of a patient’s proinflammatory and immune status. Lymphopenia indicates a decline in adaptive immunity, critical for developing targeted responses against cancer cells. This lymphocyte reduction impairs the body’s ability to recognize and respond to tumor-specific neoantigens through specific T-cell receptors [51]. Takemura et al. showed lymphopenia is an indicator of a lower objective response rate, shorter time to subsequent treatment, and poorer OS in patients with mRCC who were treated with immunotherapy-based combinations [23]. Patients who had lymphopenia and subsequently recovered their lymphocyte counts had favorable oncologic outcomes compared to those who did not recover from lymphopenia. Several other studies have demonstrated the prognostic role of peripheral blood lymphocyte levels in patients with RCC [52–54].

RCC is considered a metabolic disease, with key gene mutations affecting pathways such as glycolysis, the tricarboxylic acid cycle, glutamine metabolism, and ATP production. The Warburg effect, which is defined by the increased production of lactate and glycolysis in the presence of oxygen, is a critical component of the metabolic reprogramming of RCC. RCC cells secrete large amounts of lactate into the extracellular microenvironment, which diminishes the cytotoxic activity of T lymphocytes, reduces pro-inflammatory cytokines, and alters the antigen-presenting capacity of dendritic cells [55, 56]. Furthermore, the accumulation of HIF-α is a consequence of VHL inactivation in RCC, which enhances pathways associated with glycogen synthesis, glycolysis, and fatty acid metabolism. Increased expression of glucose transporter 1 (GLUT1) in RCC is associated with reduced CD8+ T cell infiltration, which implies that GLUT1 plays a role in the immune evasion of renal cancer cells [57]. There is a crosstalk between metabolic pathways and the immune system in RCC. Hyperglycemia and lymphopenia were individually recognized as factors that negatively impact cancer prognosis. The glucose-to-lymphocyte ratio can serve as a valuable marker, reflecting both the glucose metabolism and the systemic inflammatory status of patients.

Our study is subject to several limitations. Firstly, the study is designed retrospectively, making it particularly susceptible to missing data, selection bias, and confounding factors inherent in this type of design. Secondly, we assessed the effects of GLR in patients receiving TKI monotherapy. However, since the current first-line treatment of RCC involves immunotherapy and TKI combination therapies, it is also necessary to conduct studies on this patient group. Furthermore, the results may be influenced by patient-specific factors such as comorbidities and concurrent medications, which could impact glucose levels and lymphocyte counts. Lastly, there is no established cut-off value for GLR, which may affect the generalizability of our findings, underscoring the need for additional standardization in future research. Despite these limitations, clinical practice can efficiently utilize GLR as a simple, cheap, and noninvasive parameter. Further investigation is required to elucidate the molecular mechanisms at the molecular level and measure serum inflammatory cytokines, such as TNF-α, IL-8, and IL-6, to gain a more comprehensive understanding of the role of GLR in the prognosis of RCC.

In conclusion, our study highlights the potential predictive value of the GLR in patients with mRCC treated with TKIs. We demonstrated that high GLR levels are independent prognostic factors for PFS and OS, reflecting the interplay between metabolic dysregulation and immune response in cancer progression. While our findings are promising, the retrospective nature of our study and the lack of an established GLR cut-off value underscore the need for further prospective research, particularly in patient populations receiving current standard-of-care combination therapies.

## Data Availability

The data that support the findings of this study are available from the corresponding author upon reasonable request.
